# Heat Shock Protein 70 in Alzheimer's Disease

**DOI:** 10.1155/2014/435203

**Published:** 2014-11-06

**Authors:** Rui-Chun Lu, Meng-Shan Tan, Hao Wang, An-Mu Xie, Jin-Tai Yu, Lan Tan

**Affiliations:** ^1^Department of Neurology, Qingdao Municipal Hospital, School of Medicine, Qingdao University, No. 5 Donghai Middle Road, Qingdao 266071, China; ^2^Department of Neurology, Qingdao Municipal Hospital, College of Medicine and Pharmaceutics, Ocean University of China, Qingdao 266003, China; ^3^Department of Oncology, The Affiliated Hospital of Qingdao University, Qingdao 266003, China; ^4^Department of Neurology, The Affiliated Hospital of Qingdao University, Qingdao 266000, China

## Abstract

Alzheimer's disease (AD) is the most common neurodegenerative disease that caused dementia which has no effective treatment. Growing evidence has demonstrated that AD is a “protein misfolding disorder” that exhibits common features of misfolded, aggregation-prone proteins and selective cell loss in the mature nervous system. Heat shock protein 70 (HSP70) attracts extensive attention worldwide, because it plays a crucial role in preventing protein misfolding and inhibiting aggregation and represents a class of proteins potentially involved in AD pathogenesis. Numerous studies have indicated that HSP70 could suppress the progression of AD with *in vitro* and *in vivo* experiments. Thus, targeting HSP70 and the related compounds might represent a promising strategy for the treatment of AD.

## 1. Introduction

Alzheimer's disease (AD) is the major cause of late-life brain failure. In the current study, the data forecasted that one new case of AD is expected to develop every 33 seconds by 2050 [1]. Lost wages and healthcare expenses related to AD patients and caregivers which had been forced to be provided by families were very high [2]. Then, all efforts to find an effective disease-modifying treatment were significant. Even modest advances in therapeutic and preventive strategies that lead to small delays in the onset and progression of AD can significantly reduce the global burden of this disease [1]. Finding an effective disease-modifying treatment depends on the pathomechanistic understanding of AD. The striking pathological characteristics of AD brains are the presence of senile plaques, neurofibrillary tangles, and neuronal loss. Growing evidence has demonstrated that AD is “protein misfolding disorders” that exhibit common features of misfolded, aggregation-prone proteins and selective cell loss in the mature nervous system. A complicated array of molecular events has been implicated in the pathogenesis of AD.

Heat shock proteins (HSPs) are a class of molecular chaperones that bind with nonnative proteins and assist them to acquire native structure and thus prevent misfolding and the aggregation process during the conditions of stress [3]. HSPs have been classified into families on the basis of molecular weight including HSP100, HSP90, HSP70, HSP60, HSP40, and HSP27. Different classes of HSPs play a diverse role in influencing proper protein assembly, folding, and translocation. HSP70, HSP60, and HSP27 prevent protein aggregation and help protein folding. HSP100 releases proteins from aggregates. HSP90 plays a cardinal role in maturation and activation of number of proteins. Experimental and other indirect evidence suggested that HSPs had shown up as critical regulators of neurodegenerative processes correlated with protein misfolding in the brains of AD patients [4–6]. Therefore, HSPs are expected to have strong potential as therapeutic agents in suppressing or curing AD. HSP70 (heat shock protein 70 kDa) is an important part of molecular chaperones, which is the most conserved one and is found in almost all the intracellular compartments. HSP70 protects proteins from misfolding and assists in the refolding and reactivation of damaged proteins [7–9]. Recent studies have shown the evidence that HSP70 might have attempted to prevent aggregate formation in cells and thus suppress AD conditions [10]. HSP70 overexpression effectively protected neurons in various animal models and cellular models of AD to exert therapeutic effect [11, 12]. Therefore, these findings prompted us to investigate a potential pharmacological role of HSP70 in AD, which might bring the hope of conquering AD eventually. In this paper, we will review the current status of experimental evidence linking HSP70 function to AD pathogenesis and pharmacological therapeutics of modulating HSP70 in AD treatment.

## 2. Biochemical Characteristics of HSP70

HSP70 is the most structurally and functionally conserved proteins in HSPs. HSP70 is the most ubiquitous class of ATP-dependent chaperone protein that exerts a cytoprotective effect under a number of different conditions, which plays a central role in the cellular protein quality control and degradation systems [7, 10, 13]. In humans, the HSP70 multigene family performs function on nonnative polypeptides fueled by ATP binding and hydrolysis [14]. HSP70 binds to protein substrates to assist with their folding, degradation, transport, regulation, and aggregation prevention [13, 15–18]. They carry out these activities depending on their unique molecular structure.

HSP70 consists of two highly conserved domain structures: a 45 kDa N-terminal nucleotide binding domain (NBD) and a 25 kDa C-terminal substrate binding domain (SBD), which undergo reciprocal allosteric interactions induced by ligand binding [19]. The NBD is structurally similar to actin and hexokinase, and it consists of two lobes, each containing two subdomains [20]. These lobes form a cleft that binds ATP with a nucleotide binding cassette that is related to those in hexokinase and actin [20]. The SBD consists of a *β*-sandwich subdomain and an *α*-helical lid with the substrate binding site located in the *β*-sandwich subdomain [18]. Both the NBD and the SBD are critical for chaperone function, which are connected by a short flexible linker [19]. As one of molecular chaperones, HSP70 has multiple responsibilities during normal growth. It assists in the folding of newly synthesized proteins [13, 21, 22], the subcellular transport of proteins and vesicles [23], the formation and dissociation of complexes [24], and the degradation of unwanted proteins [25]. HSP70 which carries out these widely divergent functions arises from three different conformations. HSP70 adopts three different conformations, one in the absence of nucleotide, one with ADP bound, and one with ATP bound. Many of the functions of HSP70 depend on crosstalk between the SBD and NBD, and ATP influences substrate binding [26]. Nonnative substrates with exposed hydrophobic stretches within an accessible polypeptide backbone associate transiently with HSP70 via its SBD. ATP binding to the NBD triggers opening of the SBD binding pocket, decreasing affinity for polypeptide substrates, and accelerating both on and off rates. Reciprocally, substrate binding induces ATP hydrolysis, “closing” the SDB and thus stabilizing the substrate-HSP70 complex [27, 28]. It is this cycle of rapid but controlled binding and release of the substrate that fosters folding and assembly with partner proteins while preventing aggregation of substrates [29]. HSP70 also is essentially a protein unfolding machine, which binds and releases stretches of hydrophobic amino acids in a regulated, ATP-hydrolysis-driven cycle [30]. A global conformational change is triggered by ATP binding that expels the bound substrate; conversely, substrate binding triggers a conformational change that stimulates the hydrolysis of ATP. ATP hydrolysis in HSP70 is thought to be a major determinant of chaperone function. Cochaperones regulated their interactions with nucleotides and substrate proteins [31]. Thus, HSP70s are part of a multiprotein complex that utilizes coordinated ATPase activity and multiple cochaperone partners to shape interactions with misfolded substrates [32]. For these reasons, recent studies have investigated the fact that HSP70s have emerged as critical regulators of proteins associated with neurodegenerative disease pathologies, acting as a potential drug target.

## 3. HSP70 in the Pathogenesis of AD

### 3.1. HSP70 Plays Cytoprotective Roles in AD by Inhibition of A*β* Oligomerization

Abundant extraneuronal deposit of amyloid-beta (A*β*) is the major pathological hallmark and plays an early important pathologic role in the development of AD. Self-assembly of A*β* produces a number of distinctive structures, such as dimmers, oligomers, unstructured aggregates, and characteristic amyloid fibrils. Oligomers are believed to be the most neurotoxic and important in the development of AD [33, 34]. Many previous studies widely accepted that A*β* aggregates trigger a series of downstream events such as plaque deposition, tau hyperphosphorylation, inflammation, loss of synaptic structure and function, and death of susceptible neurons, which was deemed as “amyloid cascade hypothesis” [33, 35–37]. Theoretically, HSP70 suppresses the aggregation of A*β* to impede the pathological process of AD. Overexpression of HSP70 which inhibits the aggregation of A*β* and ameliorates the corresponding disease symptoms had been widely accepted [6, 9]. What is the molecular mechanism used by HSP70 to inhibit A*β* self-assembly? Current models suggest that HSP70 would recognize the oligomers and modify their conformation. Exposed hydrophobic regions in oligomers are “flags” to trigger HSP70 recognition and subsequent reorganization [11].

### 3.2. HSP70 Plays Cytoprotective Roles in AD by Enhancing the Clearance of A*β*

A*β* is widely considered to be the major toxic agent in the pathogenesis of AD. It is undeniable that enhancing the clearance of A*β* could suppress the progression of AD. HSP70 overexpression effectively protecting neurons from intracellular accumulation of A*β* through promoting the clearance of A*β* had been reported in a number of studies [9]. A*β* is cleared from the brain through enzyme mediated degradation, phagocytosis by microglia and astrocytes, and transport into the blood and lymph nodes. HSP70 is attributable to the stimulation of A*β* clearance through upregulation of expression of insulin degrading enzyme (IDE) and TGF-*β*1. IDE is an A*β*-degrading enzyme that motivates the clearance of A*β*. TGF-*β*1 as a key cytokine regulating the response of the brain to injury and inflammation has also been suggested to suppress the progression of AD. TGF-*β*1 stimulates A*β* clearance through activation of phagocytic microglia.

### 3.3. HSP70 Plays Cytoprotective Roles in AD by Restoring Tau Homeostasis

Neurofibrillary tangles (NFTs) composed of aggregates of hyperphosphorylated forms of the protein tau are known to be another major histopathological character in AD. Tau protein is the major neuronal microtubule-associated protein (MAP) and plays a central role in maintenance and stabilization of microtubules within axons in AD. Tau homeostasis is regulated by its expression, phosphorylation, and turnover. Tau homeostasis is disrupted, leading to hyperphosphorylation and accumulation of intracellular aggregates [38]. To date, more evidence suggests that aggregation and accumulation of the microtubule-associated protein tau are associated with cognitive decline and neuronal degeneration in AD [39, 40]. Conformational changes of tau result in its disassociation from the microtubule and self-association into aggregates. Those aggregates are the toxic form of tau which is believed to be responsible for AD pathogenesis [41]. A number of studies have suggested that neuronal and cognitive defects of AD with tauopathy can be reversed by restoring tau normal homeostasis. Understanding how to keep balance of tau homeostasis may enable identification of potential mechanisms for enhancing clearance of pathological forms of tau. Tau homeostasis is normally controlled through the action of molecular chaperones, such as HSP70. HSP70 keenly draws more and more attention of the researchers because of its potential therapeutic role in regulation of tau homeostasis. It was confirmed that HSP70 might promote tau binding to microtubules and implicate blocking tau aggregation and promoting its degradation, thereby preventing tauopathy [42–47]. HSP70 directly inhibits tau aggregation by a mechanism involving preferential associations with soluble, monomeric, and prefibrillar oligomeric tau species [48]. HSP70 assists these combinations being degraded by the ubiquitin-proteasome and autophagy system. In addition, HSP70 dramatically affects tau homeostasis by its highly homologous variants disparate capacity to clear tau. HSP70 includes the inducible form of HSP70 which is termed heat shock protein 72 (HSP72) and the constitutively expressed form of HSP70 is termed heat shock cognate 70 protein (HSC70). HSP72 and HSC70 caused distinct and even opposing effects on tau's function and stability. HSP72 can facilitate tau degradation; HSC70 actually slows tau clearance, particularly after microtubule disruption. In cellular models, inhibitors of the ATPase activity of HSP70 also have been shown to promote tau turnover and restore its homeostasis [49].

### 3.4. HSP70 Plays Cytoprotective Roles in AD by Inhibition of Neuronal Apoptosis

**Figure 1:**
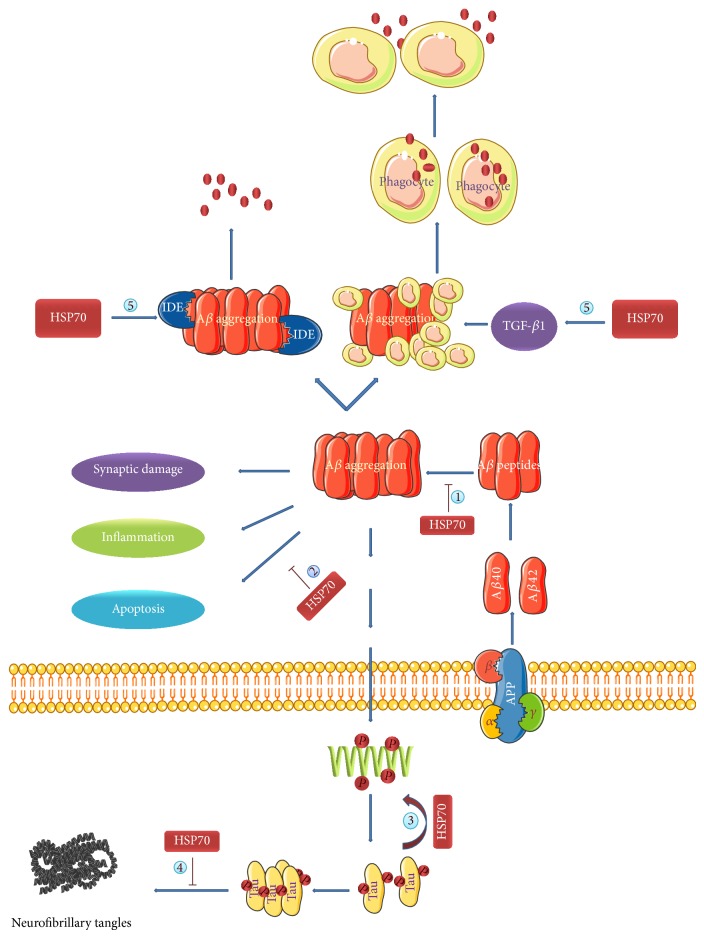
HSP70 plays cytoprotective roles in AD. (1) HSP70 recognizes A*β* oligomers and inhibits A*β* self-assembly; (2) HSP70 modulates both Apaf-1 caspase-dependent and AIF caspase-independent pathways resulting in attenuation of apoptosis; (3) HSP70 promotes tau binding to microtubules; (4) HSP70 directly inhibits tau aggregation by a mechanism involving preferential associations with soluble, monomeric, and prefibrillar oligomeric tau species; (5) HSP70 upregulates the expression of insulin degrading enzyme (IDE) and TGF-*β*, which enhance the clearance of A*β*.

Studies on postmortem tissues provide morphological and biochemical evidence that some neurons degenerate via apoptotic mechanisms in AD. One of the most momentous findings in AD brain is the large number of neuronal losses developed by pervasive DNA damage which leads to apoptosis. Many conditions accumulate in the brain that are capable of inducing apoptosis and that place neurons at continued risk.* In vivo* and* in vitro* studies have shown that A*β* accumulation caused neuronal dysfunction and synaptic and neuronal loss [50]. Neuronal apoptosis also readily initiated by oxidative insults was known to occur in AD brain. Under some conditions, glutamate and other excitatory amino acids which initiated excitotoxic damage can activate apoptosis in the development of AD. These factors may act synergistically. For example, when subthreshold levels of A*β* are coupled with subthreshold levels of excitotoxins or oxidative stress, neuronal loss is significant. Protective mechanisms probably serve to delay degeneration and maintain neurons function in AD. Mailhos et al. first confirmed that HSP70 overexpression attenuates thermal stress-induced neuronal death [51]. On the basis of these previous data, studies showed that HSP70 modulates both Apaf-1 caspase-dependent and AIF caspase-independent pathways resulting in attenuation of apoptosis and ultimately inhibition of neuronal cell death [52] (Figure 1).

### 3.5. HSP70: A Therapeutical Strategy for AD?

HSP70 as one of major molecular chaperones not only participates in the cellular protein quality control and degradation systems, but also implicates the neuroprotective response to pathological manifestations of AD [6, 11, 32, 48]. On the basis of those dramatic functions, HSP70 has increasingly attracted researchers' attention. At present, there are four main therapeutic strategies to treat AD by characteristic HSP70's functions mentioned above: induction endogenous of HSP70, utilization exogenous HSP70, efforts to differing levels of constitutively expressed HSP70, and utilization inhibitor of HSP70 ATPase.

### 3.6. Induction Endogenous of HSP70

Efforts to promote endogenous HSP70 synthesis induced in many different conditions could be therapeutically relevant for AD. Initially, induction of HSP70 during heat shock protected against AD-like hyperphosphorylation of tau in PC12 cells was reported [53]. Subsequently, induction of HSP70 by geldanamycin suppresses formation of neurofibrillary tangles by partitioning tau into a productive folding pathway and thereby preventing tau aggregation in COS-1 cells was identified [42]. Therefore, the neuroprotective effect of HSP70 in AD has received extensive attention. Based on above experimental results, YC-1 [3-(50-hydroxymethyl-20-furyl)-1-benzylindazole] was used to induce HSP70 expression, which prevented A*β*-induced cytotoxicity in PC12 cells [54]. Overexpression of HSP70 achieved by transfecting neurons with HSP70-expression plasmids significantly protects against etoposide, C2-ceramide, staurosporine, and A*β* (25–35)-induced neuronal apoptosis [52].

New advances in high throughput screening (HTS) methodology are rapidly changing the landscape of discovery in induction of HSP70. The finding that estrogen may facilitate HSP70 expression in different cells under various stress conditions was reported [55]. Recently, Hirakawa et al. indicated that HSP70 played a role in DNA protection mediated by estrogen, and the DNA protection might be involved in AD preventive effect from estrogen. Geranylgeranylacetone (GGA) has been widely considered as a nontoxic HSP-inducer although it is famous as a leading antiulcer drug [56]. Its cytoprotective, anti-inflammatory, and antiaggregation activities and induction of HSP70 expression were confirmed in animal models of various diseases. Hoshino et al. examined the effect of GGA on AD phenotypes exhibited by APP23 mice. They proposed that GGA should be considered as a candidate drug for the prevention of AD [9]. Curcumin elevated HSP90 and one of HSP70 subcellular compartments without increasing HSP mRNAs and reduced soluble tau, which showed that even after tangles had formed, tau-dependent behavioral and synaptic deficits could be corrected [57]. Celastrol is effective in inducing a set of neuroprotective HSPs in cultures derived from cerebral cortices, including HSP70, HSP27, and HSP32. It is deemed as a potential agent to counter AD [58]. Drugs mentioned above not only develop new leads for therapeutic development in AD, but also discover new chemical probes for use in understanding HSP70 biology.

### 3.7. Utilization Exogenous HSP70

Another potential remedy for AD is utilization of exogenous HSP70. Recently, intranasally administered HSP70 rapidly enters the afflicted brain regions and mitigates multiple AD-like morphological and cognitive abnormalities observed in model animals [12]. However, a long-lasting effect of exogenous HSP70 cannot be readily explained by its direct chaperone activity against misfolded AD proteins, or by indirect effects, such as the suppression of apoptosis and HSP70-mediated autophagy. Recent findings suspected that exogenous HSP70 could stimulate the innate immune response and significantly reduce the level of reactive oxygen species [59, 60].

### 3.8. Efforts to Differing Levels of Constitutively Expressed HSP70

Previous reports have shown that HSP70 includes the primary cytosolic variants, HSC70 and HSP72, and influenced tau conformation, degradation, and aggregation kinetics [61]. Some studies suggested that overexpression of HSP70 variants can facilitate tau clearance, whereas others suggested the opposing activities of HSP70 variants that HSC70 may preserve tau in a more unstructured state, perhaps facilitating its pathogenicity. Hence, using tetracycline- (Tet-) based protein chase HEK cell model to make clear these differences between HSC70 and HSP72 with regard to tau clearance. These findings suggested that HSP72 and HSC70 caused distinct and even opposing effects on tau's function and stability even if their sequences are similar. HSP72 was confirmed to accelerate tau clearance while HSC70 slows it [62]. A delicate expression level of HSP70 variants must be managed to prevent tau from accumulating to the point of being neurotoxic. Efforts to promote HSP72 expression and inhibit HSP70 could be therapeutically relevant for AD [62].

### 3.9. Utilization Inhibitor of HSP70 ATPase

**Figure 2:**
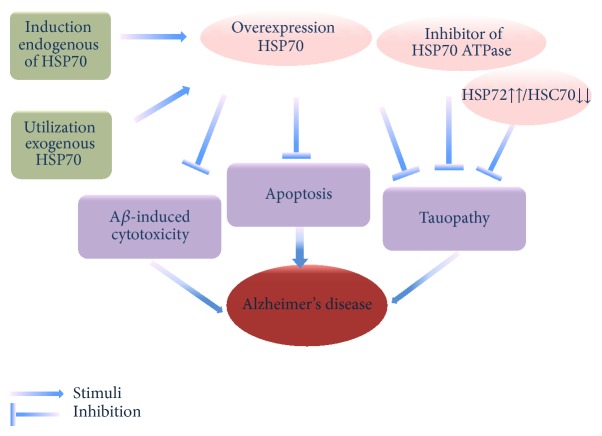
HSP70 as a therapeutic strategy for AD. Endogenous stimulus and exogenous utilization elevate the level of HSP70, which inhibit the cytotoxicity of A*β*, tauopathy in brain, and apoptosis in AD. Efforts to promote HSP72 expression and inhibit HSC70 could accelerate tau clearance, which attenuate tauopathy. Inhibitors of the ATPase activity of HSP70 reduced pathogenic tau in brain.

Inhibitors of the ATPase activity of HSP70 also come into people's line of sight because of their function as mentioned above. Methylene blue (MB), a recently described inhibitor of HSP70 ATPase, not only was shown to favor tau turnover but also was able to facilitate keeping tau homeostasis [63]. However, MB is the first generation compounds and is not selective for HSP70. MKT-077 also is one of known HSP70 inhibitors, which are HSP70 selective in cells and cannot pass the blood-brain barrier (BBB). Together these problems have limited use of MB and MKT-077 as clinical treatment. Very recently synthetic YM-08 as a neutral analogue of MKT-077 retained affinity for HSP70* in vitro* and selectively reduced pathogenic tau in brain. Thus, YM-08 may serve as a suitable chemical for further development as a CNS penetrant HSP70 inhibitor [64] (Figure 2).

## 4. Conclusions

There are numerous convincing reports to illustrate HSP70 and related compounds associate with AD pathogenesis. As mentioned previously, a large amount of evidence indicates HSP70 and related compounds have remarkable neuroprotective effects in several models of AD both* in vivo* and* in vitro*. These mechanisms between HSP70 and those pathological features of AD provoked a lot of interest to explore curing strategies. Although much evidence exists for a positive effect of HSP70 in animal and cellular models of AD, some limitations must be overcome before a therapeutic use for these proteins is established: (1) the present findings were mainly confirmed by cellular and animal experiments in AD; (2) the mode of action also was proven in different cellular and animal models of AD; and (3) some effect of increased synthesis induction of HSP70 is unpredictable, because quantitative control of the amount of HSP70 useful for a therapeutic response is difficult to determine. On the whole, further studies will be required to fully elucidate the roles of HSP70 in AD.
